# Green and Sustainable Synthesis of Silver and Gold Nanoparticles: A Stevia Leaf Extract Approach

**DOI:** 10.1002/fsn3.70698

**Published:** 2025-08-20

**Authors:** Muhammad Farhan Jahangir Chughtai, Samreen Ahsan, Adnan Khaliq, Muhammad Adil Farooq, Mir Muhammad Nasir Qayyum, Atif Liaqat, Saira Tanweer, Tariq Mehmood, Muhammad Zubair Khalid, Waseem Khalid, Yassine Jaouhari, Matteo Bordiga, Suleiman A. Althawab, Tawfiq Alsulami

**Affiliations:** ^1^ Institute of Food Science and Technology, Faculty of Food, Health Science and Technology Khwaja Fareed University Engineering and Information Technology Rahim Yar Pakistan; ^2^ Department of Agriculture and Food Technology Karakoram International University Gilgit Baltistan Gilgit Pakistan; ^3^ Department of Food Science and Technology Faculty of Agriculture and Environment, The Islamia University of Bahawalpur Bahawalpur Pakistan; ^4^ Department of Food Science, Faculty of Life Sciences Government College University Faisalabad Faisalabad Pakistan; ^5^ University Institute of Food Science and Technology The University of Lahore Lahore Pakistan; ^6^ Department of Pharmaceutical Sciences Università del Piemonte Orientale Novara Italy; ^7^ Department of Food Science & Nutrition College of Food and Agricultural Sciences, King Saud University Riyadh Saudi Arabia

**Keywords:** fourier transform infrared spectroscopy, green synthesis, silver and gold nanoparticles *Stevia rebaudiana*, transmission electron microscopy

## Abstract

Silver and gold nanoparticles were synthesized by reducing silver and gold ions using previously unexplored stevia extracts prepared with water, methanol, and ethanol as solvents. These extracts served as both reducing and stabilizing agents. The nanoparticles were characterized using ultraviolet–visible (UV–Vis) spectroscopy, which confirmed surface plasmon resonance (SPR) at 400–450 nm for AgNPs and 500–550 nm for AuNPs, with an additional peak at 650–700 nm. Zeta potential analysis revealed polydispersity index (PdI) values of 0.269, 0.328, and 0.184 for AgNPs, and 0.428, 0.366, and 0.408 for AuNPs in water, ethanol, and methanol extracts, respectively. The Z‐average particle sizes were 68.66, 163.9, and 146.4 nm for AgNPs, and 207.2, 96.27, and 160.6 nm for AuNPs. TEM images confirmed that the nanoparticles were spherical and well‐dispersed. FTIR analysis indicated that functional groups such as hydroxyl and carbonyl from the stevia extracts were involved in the reduction and stabilization of nanoparticles. This study highlights the potential of *Stevia rebaudiana* extracts as efficient, eco‐friendly agents for the synthesis of metallic nanoparticles, offering a sustainable alternative to conventional chemical and physical methods with potential applications in antimicrobial and biomedical fields.

## Graphical abstract

Stevia extracts offer a novel and environmentally friendly method for synthesizing silver (AgNPs) and gold (AuNPs) nanoparticles. The synthesized nanoparticles displayed characteristic surface plasmon resonance (SPR) in specific ranges and were confirmed to be spherical and well‐dispersed via TEM imaging. FTIR spectroscopy confirmed the affinity of stevia extracts in this green synthesis process, highlighting their strong reducing agent capabilities.

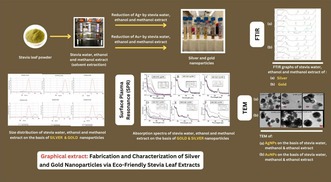

**Abbreviations**
AgNPs: Silver nanoparticles
AuNPs: Gold (III) chloride trihydrate HAuCl4.3H2O; Gold/Aurum nanoparticles
FTIR: Fourier transform infrared spectroscopy
IR: Infrared
NPs: Nanoparticles
PdI: Polydispersity index
SEEAgNPs: Stevia ethanol extract‐based silver nanoparticles
SEEAuNPs: Stevia ethanol extract‐based gold nanoparticles
SGS: zeta (Z, ζ). stevia glycosides
SMEAgNPs: Stevia methanol extract‐based silver nanoparticles
SMEAuNPs: Stevia methanol extract‐based gold nanoparticles
SPR: Surface plasmon resonance
SWEAgNPs: Stevia water extract‐based silver nanoparticles
SWEAuNPs: Stevia water extract‐based gold nanoparticles
TEM: Transmission electron microscopy
UV–VIS: Ultraviolet–visible
UV–Vis–NIR: Ultraviolet–visible–near infrared

## 1 Introduction

Nanotechnology embarks on a ground‐breaking journey in technological advancement, delving into the manipulation of matter at the scale of nanometers, promising a wide range of unprecedented possibilities. Nowadays, product research and development in the field of nanotechnology has gradually increased, owing to the novel and useful properties of nanoscale materials (Khatami, Sarani, et al. 2019). Nanotechnology development is a disruptive technology that provides efficient and sustainable nano‐bio interaction alternatives (Nasrollahzadeh et al. 2019).

In this regard, nanotechnology is a versatile tool used in various antibacterial agents, composite materials, catalysts, and electrical devices. It encompasses a broad expression that exemplifies the pinnacle of man's insatiable desire for knowledge with practical application (Ahmad et al. 2023). The unique physicochemical features of nanoparticles (NPs) have led to their applications in different fields. Indeed, many metal nanoparticles and metal oxides have been successfully manufactured. The extraordinary surface‐to‐volume ratios of nanoparticles constitute a key feature. This elevated ratio is due to the smaller particle size, which improves their efficacy in biological contexts (Khatami, Alijani, et al. 2019).

Even though several chemical and physical approaches are used in the production of nanoparticles, it is important to point out that these procedures are frequently aligned with the formation of dangerous substances that possess inherent toxicity (Hemlata et al. 2020). The traditional physical and chemical methods for synthesis of nanoparticles need toxic solvents, high temperatures, and dangerous reducing agents including hydrazine, sodium borohydride, or organic amines. These processes not only endanger human health but also the environment by releasing hazardous waste and consuming a lot of energy. Conversely, green synthesis approaches are environmentally friendly by using renewable plant‐based materials and using mild conditions for temperature, solvent, and pressure (Nagime et al. 2024). Plant‐mediated synthesis, termed “green synthesis” is gaining popularity due to its low toxicity, commercial viability, environmental friendliness, and time saving. Green synthesized nanoparticles that are made from extracts of different plants have huge biomedical applications, and that is because of their cytotoxic and bactericidal properties (Daghestani et al. 2020). Although the production of metallic nanoparticles from plant extracts has been documented in several plant species—including geranium, neem, herb aloe, lichen, pear fruit, *Mangifera indica* , and *Magnolia kobus* —there is still a great deal of interest in this area due to the variety and high efficacy of plants for the extraction of nanoparticles of various forms (Sadeghi et al. 2015). Aurum and silver particles of nanoscale have piqued the interest of many scientists who are fascinated by the subject matter due to their biological potential (Sadeghi et al. 2015). The silver nanoparticles (AgNPs) have potential as potent antibacterial agents to target antibiotic‐resistant pathogens by incorporating oxidative stress and morphological changes in pathogenic microbial strains that are small in size and great in efficacy (Bhat, Alonazi, et al. 2024). Another group of researchers has also confirmed tremendous antimicrobial activity of silver nanoparticles from citrus waste against different pathogenic strains of Gram‐positive and Gram‐negative bacteria (Bhat, Al‐Dbass, et al. 2024). Thus, several funded projects have focused on the sustainably sourced production of silver nanoparticles (AgNPs) and gold/aurum nanoparticles (AuNPs) using plant leaves. Nonetheless, there is an undiscovered avenue for the synthesis of AgNPs in which wild and native species are harnessed for their intrinsic anti‐cancer and anti‐bacterial activity. Stevia ( *Stevia rebaudiana* Bertoni) emerges as a prominent potential in this context due to the wide range of species that find relevance in human ingestion. Furthermore, stevia's significant anti‐cancer properties—notably against a variety of cancer cell lines such as kidney, brain, tumor, and melanoma—increase its potential application in the field (Hemlata et al. 2020). *Stevia* is native to South America, which is a medicinal plant, and traditionally it has also been used as a natural sugar substitute due to its intensely sweet leaves. Its epidemiological properties are due to its bioactive compounds, such as stevioside, rebaudioside A, flavonoids, phenolic acids, alkaloids, terpenoids, and tannins. These phytochemicals show a broad spectrum of biological activities including antioxidant, antimicrobial, anti‐inflammatory, anticancer, and antidiabetic effects (Myint et al. 2023). Recent studies have highlighted the redox potential of these compounds, making *Stevia* an ideal candidate for green synthesis of metal nanoparticles where both reduction and stabilization are required (Chakma et al. 2023).

Unlike other plants whose infusions are frequently used, sun‐dried leaves of *S. rebaudiana* serve a particular function in synthesizing gold nanoparticles (Mittal et al. 2013). There have been assertions that the involvement of reducing sugars (aldoses) and terpenoids in stevia is important in the process of reducing gold and silver ions and enabling the creation of nanoparticles (Ahmad et al. 2023) This novel methodology employs plant extracts using different solvents, including water, methanol, and ethanol, to aid in the reduction and stabilization of these nanoparticles, presenting an environmentally benign and biocompatible method with a wide range of uses (Ikram 2015). Plant‐mediated NP production is preferred over other biological methods since there is no disruption in cell culture preservation and maintenance. In comparison to microbes, plant‐mediated synthesis is a simple one‐step process with no mutation hazards. Furthermore, filtration and purification can be easily scaled up for substantial NP production (Veerasamy et al. 2011). Moreover, nanoparticles have a wide range of applications such as antioxidant, anticancer, antimicrobial, biosensors, and in food packaging. The modern nanotechnology applications in food science comprise the innovations in food packaging, functional foods development, and formation of delivery systems for bioactive compounds (Milinčić et al. 2019). Stevia's deployment in nanoparticle production demonstrates the efficacy of sustainable and biologically driven processes in nanotechnology. As this field's scrutiny advances, a harmonic union of natural extracts and advanced materials can be obtained, paving the way for novel solutions to modern difficulties. The burgeoning collaboration between science and nature highlights the boundless possibilities that unfold when scientific endeavors embrace the innate wisdom of the natural world (Sadeghi et al. 2015). Moreover, nanoparticles coating with plant material are less harmful as compared to pure silver nitrate base nanoparticles (Bhat et al. 2019).

Stevia leaf extract has strong antioxidant potential due to the presence of bioactive moieties in it including terpenoids, flavonoids, polyphenols, and glycosides such as stevioside and rebaudioside‐A that show antioxidant properties. These compounds play a duplex role during the synthesis of nanoparticles, as reducing agents by donating electrons to reduce metal ions (e.g., Ag^+^ to Ag^0^ or Au^3+^ to Au^0^); and as capping agents, by stabilizing the surface of newly formed nanoparticles by binding via hydroxyl, carbonyl, or amine groups. This prevents agglomeration and helps to maintain discrete particles and promotes consistent morphology of nanoparticles (Myint et al. 2023).

Silver based nanoparticles have unique size about 1–100 nm and have used as antimicrobial agent in health and food industries (Al‐Dbass et al. 2022). If we talk about biological activity of stevia nanoparticle studied previously that Stevia‐mediated silver and gold nanoparticles exhibit antimicrobial activity due to their small size, enabling strong interaction with microbial membranes. The release of metal ions and generation of reactive oxygen species disrupt cellular functions and lead to microbial death. Additionally, stevia‐derived polyphenols capping the nanoparticles enhance their stability and contribute to their bioactivity (Wang et al. 2014). That's why they act as a nanomedicine as drug delivery vectors, theragnostics and anti‐cancer (Kut et al. 2022). Silver based nanoparticles by using different other plant extracts *Pulicaria vulgaris* Gaertn. such as have also showed antifungal activities and antioxidant activity and proved the convenient biomedical applications (Hanna et al. 2023), Likewise, another silver based *Lallemantia royleana* leaf extract based silver nanoparticles exhibit antioxidant and antimicrobial, anti‐inflammatory, anti‐arthritic, cytotoxic, and catalytic activities (Hussain et al. 2024). Moreover, Gold nanoparticles prepared from *Nepeta bodeana* leaf extract have also showed anticancer, antidiabetic, anti‐inflammatory, and a significant antimicrobial potential (Nayem et al. 2020).

Stevia‐mediated synthesis embodies this approach, providing an affordable, scalable, and environmentally sustainable alternative that aligns with principles of green chemistry and promotes sustainable development. The current research study aims at using a green approach for the synthesis of silver and gold nanoparticles in different solvents (water, methanol and ethanol). The stevia extract was studied for antioxidant, total flavonoids, and phenolic content. Then synthesized nanoparticles were further characterized by ultraviolet–visible–near infrared (UV–Vis–NIR) spectrometry, transmission electron microscopy (TEM) and fourier transform infrared (FTIR) spectroscopy. This study pioneers the use of *Stevia rebaudiana* leaf extracts in three solvents to synthesize silver and gold nanoparticles, enabling direct efficiency comparisons. It integrates physicochemical characterization with antioxidant analysis, revealing how solvent polarity affects nanoparticle morphology, size, and stability. These insights advance plant‐mediated synthesis and highlight Stevia's role as a multifunctional agent in green chemistry.

## 2 Material and Methodology

### 2.1 Steviosides Extraction

*Stevia rebaudiana* leaves were procured from Ayub Agricultural Research Institute (AARI), Faisalabad, Pakistan. Steviosides were extracted from stevia leaf powder by different extraction solvents, including water, ethanol, and methanol. The procedure was divided into initial extraction, secondary extraction, filtration and concentration, centrifugation and sterilization, and finally storage until analysis. All extractions were performed in triplicate to ensure reproducibility. In the initial extraction, 5 g stevia powder were added separately to 100 mL of each solvent in separate glass flasks: distilled water (Kroger, Cincinnati, Ohio, USA), methanol (HPLC grade, Fischer Chemical, Pittsburgh, Pennsylvania, USA), and ethanol (200 PROOF, Anhydrous, USP Specs Decon Labs Inc., King of Prussia, Pennsylvania, USA) with subsequent heating at 30°C for 30 min on a hot plate (Thermo Scientific, Waltham, Massachusetts, USA). Then, the secondary extraction extracted sample was shifted from glass flasks to an Eppendorf tube (Eppendorf, Burlington, Massachusetts, USA) and stirred in a Reciprocal Shaking Bath (Thermo Fisher Scientific, Washington, USA) at 45°C for 30 min. After that, it was kept at room temperature for 60 min and filtered with Whatman filter paper 41. The extract was then concentrated by heating until it reduced to half its volume and then centrifuged (VWR Clinical 100 Laboratory, avantor, Radnor, Pennsylvania, USA) at 10°C for 2500 rpm for 10 min. All of these solutions were microfiltered (Millipak, Merck, Burlington, Massachusetts, USA) in microfilters (0.22 μm) porous sizes before analysis to remove any solid residue and stored at 4°C (Wang et al. 2014). After extraction, filtration, and concentration, the extract was dried (by evaporating the solvent) and the dry residue was weighed; the sample at the start was 5 g, and after drying, the yield of water extraction obtained was 0.45 g of dried extract, 0.62 g from ethanol, and 0.88 g from methanol.

Extract yield was calculated by using the following equation:

$$\text{Extractive Yield}=\frac{\text{Weight of dried extract}\;\left(g\right)}{\text{Weight of dried material}\;\text{used}\;\left(g\right)}\times 100$$

#### 2.1.1 Antioxidant, Total Flavonoids, and Phenolic Content of Stevia Extract

The DPPH free radical scavenging activity of Stevia extract was measured by following the procedure described by Chakma et al. (2023) and Ferric reducing‐antioxidant power (FRAP) assay was done, and the stock solution included 10 mM TPTZ (2, 4, 6‐tripyridyl‐s‐triazine) solution, and the absorbance was taken at 593 nm by following the detailed procedure explained by Milinčić et al. (2019) and ABTS was measured by using Trolox Equivalent reagent by following the protocol of Kut et al. (2022) (μM TE/L). TPC were estimated by using Gallic acid (GAE) as a standard curve, and GAE stock solution was prepared by using methanol; the method was followed by Hanna et al. (2023). The total flavonoids were calculated by following the detailed protocol of Hussain et al. (2024).

### 2.2 Fabrication of Aurum (AuNPs) and Silver (AgNPs) Nanoparticles

The gold (III) chloride trihydrate HAuCl_4_.3H_2_O (520918‐1G Sigma Aldrich, Saint Louis, USA) (molecular weight of 393.83 g/mol) solution was prepared with distilled water, and pH was 5.2 ± 0.2. Subsequently, an equivalent volume of clarified stevia leaf extract was introduced to a 0.1 mM AuCl4 solution (Sadeghi et al. 2015). Then the color was slightly changed from brownish to reddish.

**FIGURE 1:**
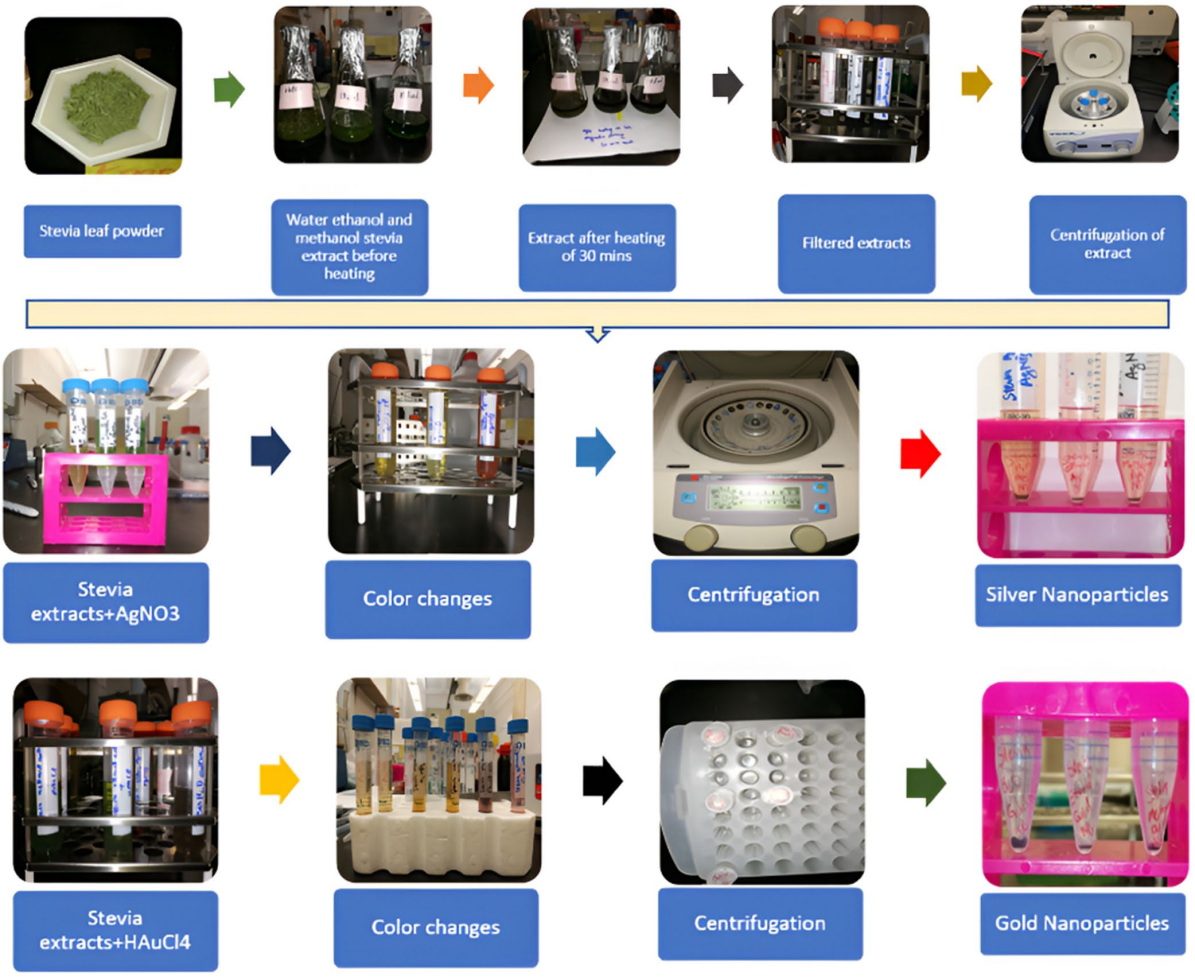
Synthesis of nanoparticles from stevia leaf powder.

To produce AgNPs, 9 mL of 1 mM AgNO_3_, p.a. (ACROS FW 169.87, d 4.35, USA) aqueous solution and pH 6.3 ± 0.2 were mixed with 1 mL of extract and stirred for 4 h (Nayem et al. 2020) before analysis (Figure 1). For aqueous, ethanol, and methanol extracts in silver nitrate reaction, the color changed from yellow to brownish, which shows the formation of AgNPs after 30 min (Laguta et al. 2019).

### 2.3 Surface Plasmon Resonance (SPR)

Sample's absorption was evaluated by using a UV–VIS–NIR Spectrometer Jasco (V‐570, Halifax, Nova Scotia Canada). The spectra were obtained from 3 mL of each sample of AgNPs and AuNPs in a 1 cm path length quartz cuvette with a blank (solvent) and silver and gold solution, and absorption was checked after 5–2400 min, and in some samples, up to 3200 min (Nayem et al. 2020).

### 2.4 Particle Size

The particle size distribution of the nanoparticles was measured by using the Zetasizer Nano‐ZS ZEN 3600 (Malvern Instruments, Worcestershire, UK) and the data was analyzed by using Zetasizer Software Version 7.13 (Katta and Dubey 2021).

### 2.5 Transmission Electron Microscopy (TEM)

The nanoparticles were characterized by using transmission electron microscopy (FEI/PHILIPS CM‐100 TEM, Hillsboro, Oregon United States). A droplet of the samples prepared by green synthesis was applied onto a lacy carbon‐coated copper grid. The samples were prepared by using a copper grid that was coated with carbon. The sample was put on this grid and then dried. Images were attained using a Gatan US1000 2 K CCD camera on a FEI Tecnai G2 20 electron microscope equipped with a LaB6 source and operating at 200 kV. Images were captured at a magnification of 100,000×, with a scale bar corresponding to 10 nm. Particle size analysis was conducted using ImageJ software, where the scale was calibrated individually for each image (Baláž et al. 2021).

### 2.6 Fourier Transform Infrared Spectroscopy (FTIR)

The FTIR spectra of all samples were recorded by Nicolet Magna‐IR Spectrometer 550 (SKU: 8356‐30‐1000, US) to characterize various functional groups and gather sufficient information about green synthesized stevia nanoparticles. Scans were taken in a range of 400–4000 cm^−1^m^−1^ (Wulandari et al. 2015).

### 2.7 Statistical Analysis

The data was analyzed using Statistix 8.1 software; the values were in triplicate, and statistical analysis was performed using complete randomized design (CRD) by following Tukey's post hoc test to determine significant differences between groups. A confidence interval of *p* < 0.05 was considered statistically significant.

## 3 Results and Discussion

### 3.1 Extractive Yield

The extractive yield refers to the amount of material obtained from a plant after solvent extraction, and it provides insight into the efficiency of each solvent in extracting bioactive compounds. From water extraction, 0.45 g of dried extract was obtained, and from ethanol, 0.62 g, while the highest was from methanol, and that was 0.88 g.

### 3.2 Antioxidant, Total Flavonoids, and Phenolic Content of Stevia Extract

The stevia extract was studied to know the effect of extraction method on antioxidant potential, flavonoid and phenolic content. The results are given in Table 1 depicting that methanol extraction have highest potential such as ferric reducing antioxidant power (324.15 μmol Fe^2+^/g). The ability of Stevia to scavenge free radicals by using different extracts was also analyzed using ABTS• + scavenging assay ABTS was 51.40 (μM TE/L) and by DPPH (% reduction) was 52.87 The maximum total flavonids contents (TFCs) have noticed in stevia methanol extract was 27.14 mg CE/g and minimum in stevia water extract (19.88 mg CE/g) and in stevia ethanol extract it was found out to be 23.30 mg CE/g. The difference in the TFCs from Stevia leaves powder depended upon the extraction solvent, their solvation properties, time, temperature and technique given for the extraction. Similar trend was noticed in total phenolic content (TPC) was 32.45 (mg GAE/g) in stevia methanol extract likewise, 28.21 mg GAE/g in stevia ethanol extract and 24.24 mg GAE/g was of stevia water extract. The results were in relation to (Hanna et al. 2023) phenolic they reported presence of total phenolic and flavonoid content in their research on s*tevia* extract. It shows the great redox potential of stevia extract due to their antioxidant and phenolic compounds to scavenge free radicals. It has reported earlier that stevia as an alternate of sugar shows strong antioxidant potential owing to the presence of various compounds with significance such as phenolic compounds, flavonoids, stevioside and rebaudioside‐A and phenolic acids (Ameer et al. 2020). Another group of researcher reported antioxidant potential of stevia leaves extract and total phenolic contents such as 7.8–21.63 mg GAE/g of and total flavonoid contents 31.37–70.41 mg QE/g, EC_50_ values of DPPH 0.41–1.10 and ABTS from 0.82 to 2.96 mg/mL (Chakma et al. 2023).

**TABLE 1:** Antioxidant, total flavonoids, and phenolic content of stevia extract.

Extraction method	FRAP (μmol Fe^2+^/g)	ABTS (μM TE/L)	DPPH (% reduction)	TFC (mg CE/g)	TPC (mg GAE/g)
Stevia methanol extract	324.15 ± 1.38^a^	51.40 ± 1.62^a^	52.87 ± 0.28^a^	27.14 ± 0.16^a^	32.45 ± 0.61^a^
Stevia ethanol extract	294.45 ± 0.90^b^	41.12 ± 2.19^b^	47.15 ± 0.26^b^	23.30 ± 0.63^b^	28.21 ± 0.28^b^
Stevia water extract	236.57 ± 1.37^c^	25.79 ± 0.97^c^	42.41 ± 1.05^c^	19.88 ± 0.11^c^	24.24 ± 0.48^c^

*Note:* Values expressed are means ± standard deviation of three values. a‐c means in column with different superscripts differ (*p* < 0.01).

### 3.3 Surface Plasmon Resonance (SPR)

**FIGURE 2:**
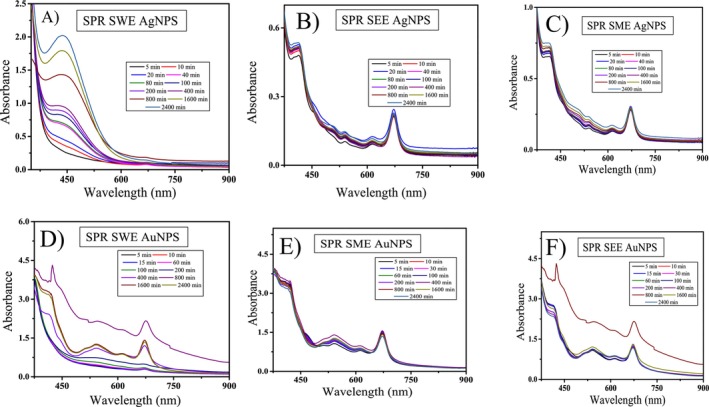
SPR plot for UV–Vis absorption spectra of, (A) *Stevia* water extract‐based silver nanoparticles (B) *Stevia* ethanol extract‐based silver nanoparticles (C) *Stevia* methanol extract‐based silver nanoparticles (D) *Stevia* water extract‐based gold nanoparticles (E) *Stevia* ethanol extract‐based gold nanoparticles (F) *Stevia* methanol extract‐based gold nanoparticles.

The surface plasmon resonance shows the ultraviolet–visible (UV–VIS) absorption peak assigned to a band of silver and gold nanoparticles of stevia extracts of water, ethanol, and methanol fabricated by reduction of AgI ions and Au^3+^ ions. The reduction mechanism involves electron donation from hydroxyl and carbonyl groups of polyphenols and terpenoids present in extract of stevia plant, converting metal ions into zero‐valent nanoparticles while the biomolecules serve as both reducing and capping agents and convert int. zerovalent metallic form such as Ag^+^ to Ag^0^ and Au^3+^ to Au^0^ (Yadav et al. 2024). UV–Vis spectra of Gold (HAuCl_4_) and silver (AgNO_3_) salt solutions alone, and Stevia extracts (water, ethanol, and methanol) without metal salts, as control samples to compare with the spectra of synthesized nanoparticles are given in Figure 2A–E. These spectra showing that the metal salts and extracts alone do not exhibit any significant SPR peaks in the 400–700 nm range. In comparison to SPR peaks appear only after nanoparticle formation, confirming the successful synthesis of gold and silver nanoparticles.

The peaks show confirmation of the nanoparticles and the presence of more than one peak is probably due to the formation of nanoparticles of different shapes and sizes (Begum et al. 2009). Figure 2A–C show detailed graphics of peak absorbance and wavelengths throughout time for stevia water extract‐based silver nanoparticles (SWEAgNPs), stevia methanol extract‐based silver nanoparticles (SMEAgNPs), stevia ethanol extract‐based silver nanoparticles (SEEAgNPs), respectively. Likewise, in Figure 2D–F are detailed graphics of peak absorbance and wavelengths throughout the time of stevia water extract‐based gold nanoparticles (SWEAuNPs), stevia methanol extract‐based gold nanoparticles (SMEAuNPs), stevia ethanol extract‐based gold nanoparticles (SEEAuNPs), respectively.

The peak absorbance was recorded from 5 to 3200 min and plotted against wavelength. The main peak wavelengths for stevia silver water extract are demonstrated in Figure 2A. The findings indicated that the initial peak manifested at 439.5 nm and it was the only highest peak with maximum absorbance (Abs) value of 2.31882 followed by a decline in slope. As time progressed, the rate of change diminished, and concurrently, the absorbance exhibited an upward trend. UV–Vis absorption spectrum was started to record after 5 min and maximum absorbance at 450 nm showed the formation of Ag nanoparticles. Figure 2B shows peaks at an absorbance of 412.5 nm (Abs was 0.4736), 537.5 nm (Abs was 0.0968), 613.5 nm (Abs was 0.06495), and 671.5 nm (Abs was 0.17443) after that, the slope showed a decreasing trend. Figure 2C depicts the highest peak at 402 nm (Abs was 0.75105) then at 520.5 nm (Abs was 0.22648) and 613 nm (Abs was 0.13181) wavelength; after that, it showed maximum. Overall, the stevia silver nanoparticles depicted the maximum absorbance starting from 400 to 600 nm and the strongest peaks with maximum absorbance were also in this range as shown in Figure 2A–C However, the absorption difference could also be related to the difference in the shape of silver nanoparticles by uneven electron distribution on the metal surface.

Silver shows surface plasmon resonance at 440–460 nm, and it depends upon the size of nanoparticles (Ismail et al. 2019), after which the rate of change is reduced, and finally, a saturation is started to be observed after 500 nm. Afterward, another peak was observed at 650–700 nm. Katta and Dubey (2021) also observed the broader peak at 420 nm, which was the corresponding SPR of AgNPs. At this point of the SPR, it is presumed that collective oscillation of electrons occurs. On the other hand, Baláž et al. (2021) have also reported the SPR of AgNPs, which was usually observed at 400–470 nm. Likewise, Mehata (2021) worked on green synthesized silver nanoparticles with ginger rhizome and reported that the highest absorption at 407 nm was considered a strong SPR, which occurs when the interaction of free electron oscillation of AgNPs is in resonance with the frequency of light interrelated. Another researcher group reported a sharp peak of SPR at 400 nm, assuringthe formation of AgNPs (Almukaynizi et al. 2022).

Gold nanoparticles showed strong peaks in stevia gold water extract at 370.5 nm (Abs was 3.92611), 524.5 nm (Abs was 0.74514), and 668 nm (Abs was 0.49237) (Figure 2D). Gold nanoparticles of stevia methanol extract show peaks at 355 nm (Abs was 4.10543), 420.5 nm (Abs was 3.07537), 541 nm (Abs was 1.22772), and 610.5 nm (Abs was 0.87251) (Figure 2E). Finally, for stevia ethanol extract, gold nanoparticles peaks were shown at 351.5 nm (Abs = 4.15085), 370.5 nm (Abs = 3.69502), 542 nm (Abs = 1.09178), and 612 nm (Abs = 0.88329) (Figure 2F). However, the absorption difference could also be related to the difference in the shape of gold nanoparticles due to uneven electron distribution on the metal surface.

Findings of the current study are also relevant to previous works that reported that gold nanoparticles SPR peak at 535 nm was close to the recent findings' first peak (ElMitwalli et al. 2020). Other studies confirmed the synthesis of spherical gold nanoparticles by forming peaks in the range of 540–550 nm (Xin Lee et al. 2016). Among all the results of SPR from different solvents nanoparticles the strongest peaks were observed in stevia water extract which were also confirmed by previous study (Sirry et al. 2020).

### 3.4 Size Distribution of Nanoparticles

Polydispersity index (PdI) and size distribution by intensity, the Z‐average, which is a measure of the average particle size in a sample that represents the mean hydrodynamic diameter of the particles, have been measured to evaluate the stability of AuNPs and AgNPs prepared with stevia water, ethanol, and methanol extracts. The PdI value of all particles was greater than 0.04, showing that it was not a single particle size but multiple particle sizes that were seen in all samples, as shown in Table 2. The PdI values of AgNPs from stevia water extract were 0.269, whereas the PdI of AgNPs from ethanol extract was 0.328; finally, the PdI of AgNPs from methanol extract was 0.184. Furthermore, their Z‐average size was 68.66, 163.9, and 146.4 nm in stevia water, ethanol, and methanol extract AgNPs, respectively. In terms of the charge of nanoparticles, they were all positively charged, showing a biological adhesion feature and strong bonding of stevia extract with silver chloride solution to synthesize nanoparticles. This also displays their stronger affinity and improved surface area. The agglomeration peak in all graphics was disregarded, and emphasis was only on the larger peak specifically for the size evaluation of nanoparticles. The nanoparticle sizes observed in this study showed that smaller nanoparticles (< 100 nm) from the methanol and ethanol extracts have a great potential in nano‐medicine due to drug delivery and cellular uptake across biological membranes. However, slightly larger but very well‐dispersed nanoparticles, such as gold nanoparticles from the water extract can be favorable for biosensing, wound healing, and in food packaging systems due to their controlled release mechanism (Hoshyar et al. 2016).

**TABLE 2:** Zetasizer characteristics of silver and gold nanoparticles synthesize from stevia.

Nanoparticles	Characteristics	Values	Peaks	Size (d.nm)	% Intensity	St. Dev (d.nm)
SWEAgNPs Result Quality: Good	Z‐Average (d.nm)	68.66	Peak 1	96.02	94.6	2.68
	Pdl	0.269	Peak 2	13.23	4.4	3.566
	Intercept	0.891	Peak 3	4.964	1.1	0.8633
SEEAgNPs Result Quality: Good	Z‐Average (d.nm)	163.9	Peak 1	207.0	94.2	96.85
	Pdl	0.328	Peak 2	38.46	4.2	8.209
	Intercept	0.892	Peak 3	5229	1.6	457.5
SMEAgNPs Result Quality: Good	Z‐Average (d.nm)	146.4	Peak 1	183.2	991	86.31
	Pdl	0.184	Peak 2	30.43	0.9	5.179
	Intercept	0.912	Peak 3	0.00	0.0	0.000
SWEAuNPs Result Quality: Good	Z‐Average (d.nm)	207.2	Peak 1	232.0	75.4	131.8
	Pdl	0.428	Peak 2	2676	21.2	1254
	Intercept	0.887	Peak 3	31.59	3.5	8.332
SEEAuNPs Result Quality: Good	Z‐Average (d.nm)	96.27	Peak 1	128.4	92.0	60.71
	Pdl	0.366	Peak 2	16.52	5.4	5.620
	Intercept	0.900	Peak 3	5028	2.6	594.3
SMEAuNPs Result Quality: Good	Z‐Average (d.nm)	160.6	Peak 1	174.5	79.0	92.21
	Pdl	0.408	Peak 2	2653	18.7	1258
	Intercept	0.887	Peak 3	22.13	2.4	5.945

As shown in Table 2, the PdI values of AuNPs from stevia water extract was 0.428, and PdI of AuNPs from ethanol extract was 0.366, while PdI of AuNPs from methanol extract was 0.408. Additionally, their Z average size was 207.2, 96.27, and 160.6 nm in stevia water, ethanol and methanol extract AuNPs, respectively. In SWEAuNPs, the peak 1 was 232.0 nm with an intensity of 75.4%, whereas in AuSEENPs the peak 1 was 128.4 nm and intensity was 92%, and, finally, in SEEAuNPs the peak 1 was 174.5 nm with 79% intensity. Although two other smaller peaks were showing agglomeration they were ignored because we are concerned with the size of particles in the highest peak (Ali Dheyab et al. 2020).

In terms of nanoparticle charge, they were all positively charged and showed a biological adhesion feature and strong bonding of stevia extract with aurum chloride solution to synthesize nanoparticles. This also shows their stronger affinity and improved surface area. The agglomeration peak present in all graphs was ignored, and the larger peaks were highlighted to evaluate the size of nanoparticles (Hassani et al. 2019). The positive zeta potential values ranging from +20 to +35 mV suggest the moderate colloidal stability that is required for biological interactions and shelf stability in commercial formulas. Moreover, the higher the stability of nanoparticles resists agglomeration and increases their activeness time in aqueous environments, which enhances their suitability in active food packaging, where extensive antimicrobial activity is required (Omerović et al. 2021).

**FIGURE 3:**
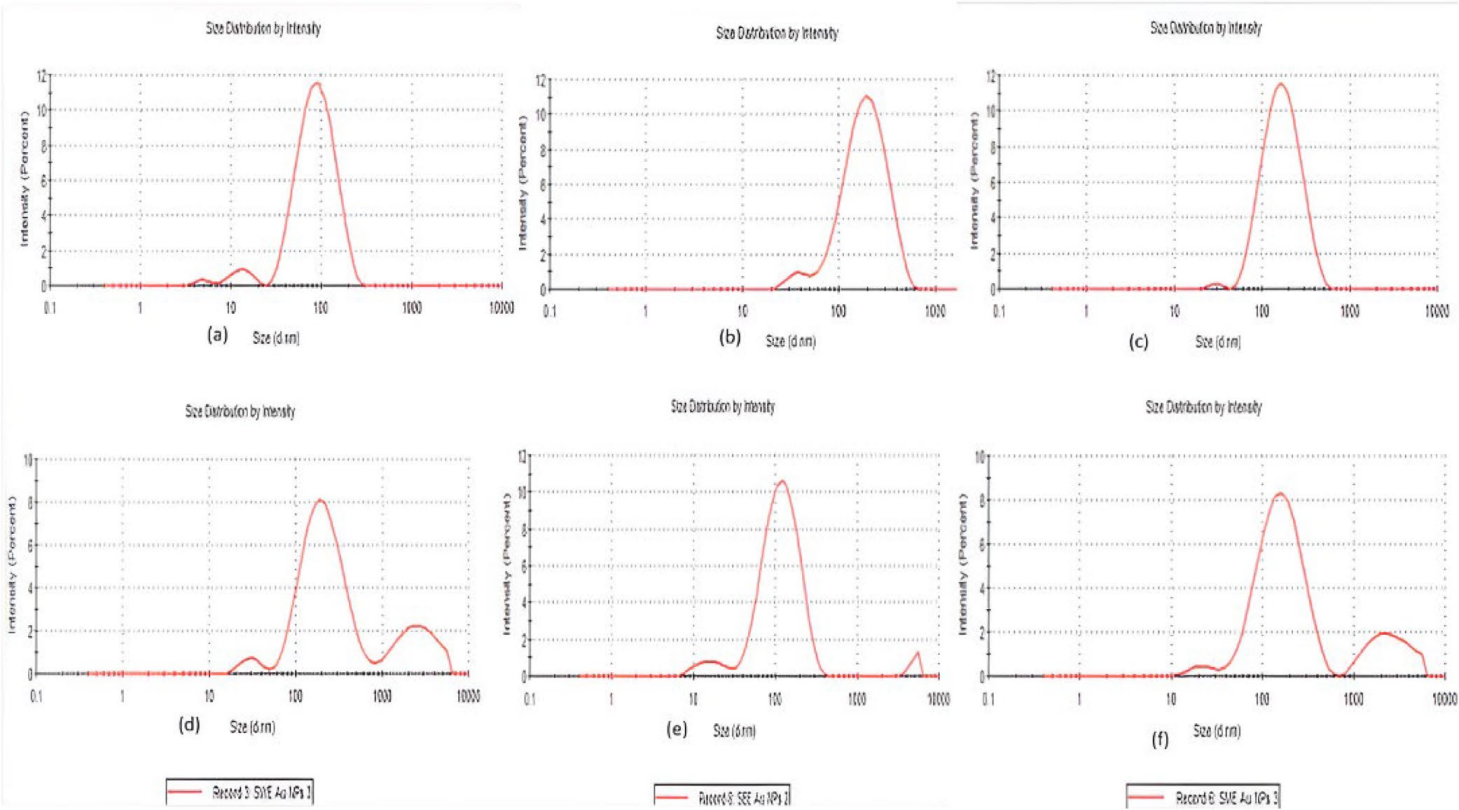
(A) Size distribution of Stevia Water extract Silver Nano Particles. (B) Stevia Methanol extract Silver Nano Particles. (C) Stevia Ethanol extract Silver Nano Particles. (D) Stevia Water extract Aurum Nano Particles. (E) Stevia Methanol extract Aurum Nano Particles. (F) Stevia Ethanol extract Aurum Nano Particles.

The results of our findings are similar to a previous study (Singh et al. 2018), in which they worked on green synthesized Ag and Au nanoparticles by leaf extract of *Euphrasia officinalis* . They reported that the average particle size of silver nanoparticles was 40.37 ± 1.8 nm with a PdI of 0.382. That is very close to our results as the average particle size and PdI of silver nanoparticles were, respectively: 68.66 nm and 0.269 in SWEAgNPs; 146.4 nm and 0.184 in SEEAgNPs; and 163.9 nm and 0.328 in SMEAgNps (Figure 3A–C, respectively). Likewise, they demonstrated that gold nanoparticles were 49.72 ± 1.2 nm and PdI was 0.484. In comparison to the current work, the average particle size and PdI of golden nanoparticles were, respectively.

207.2 nm and 0.428, in SWEAuNps; 160.6 nm and 0.408, in SMEAuNPs; and 96.27 nm and 0.366, in SEEAuNps (Figures 3A and 5B,C, respectively). Hence, the present study provides insights into the stability of nanoparticles (NPs) based on the range of zeta potential values. It highlights the noteworthy stability of NPs in water, ethanol, and methanol solvents employed in the stevia extraction process.

### 3.5 Transmission Electron Microscopy (TEM)

**FIGURE 4:**
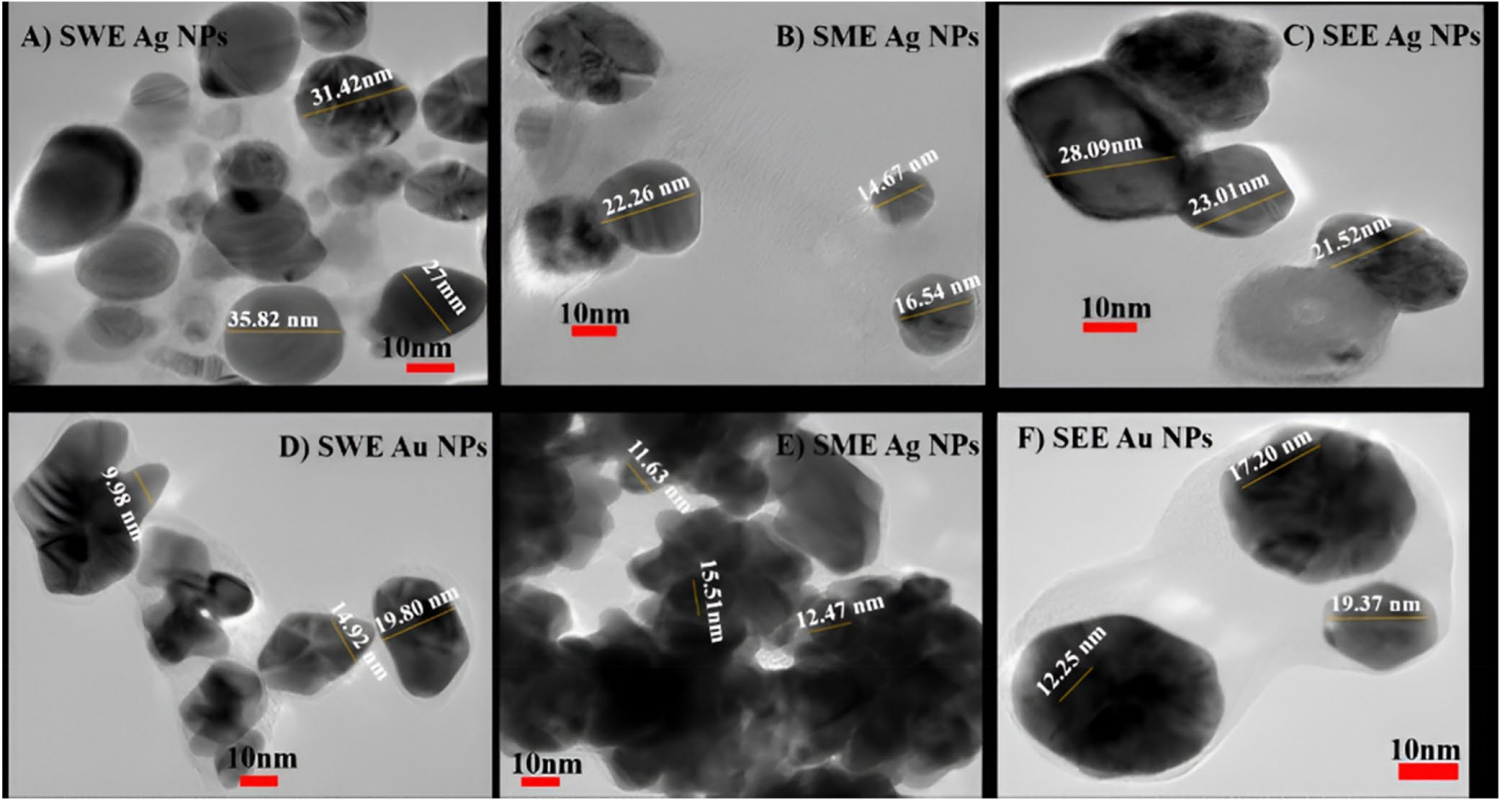
Transmission electron microscopy (TEM) of (A) *Stevia* water extract, (B) *Stevia* methanol extract, (C) *Stevia* ethanol extract in AgNO_3_ solution, (D) *Stevia* water extract, (E) *Stevia* ethanol extract, (F) *Stevia* ethanol extract in AuCl_4_ solution.

**FIGURE 5:**
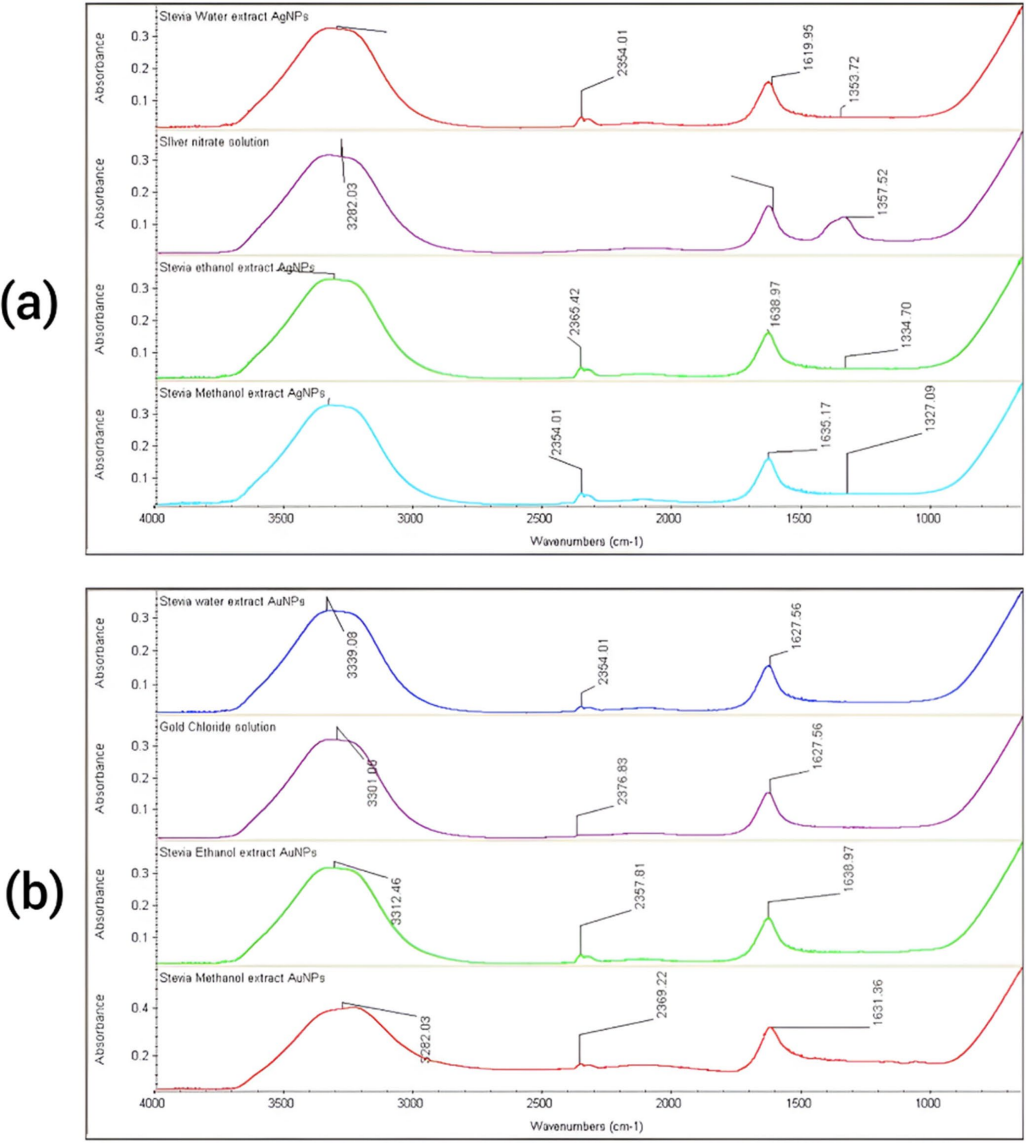
(A) FTIR graphs of Stevia Water, Ethanol, and Methanol Extract Silver nanoparticles, (B) FTIR graphs of Stevia Water, Ethanol, and Methanol Extract Gold nanoparticles Stevia Ethanol Extract.

**FIGURE 6:**
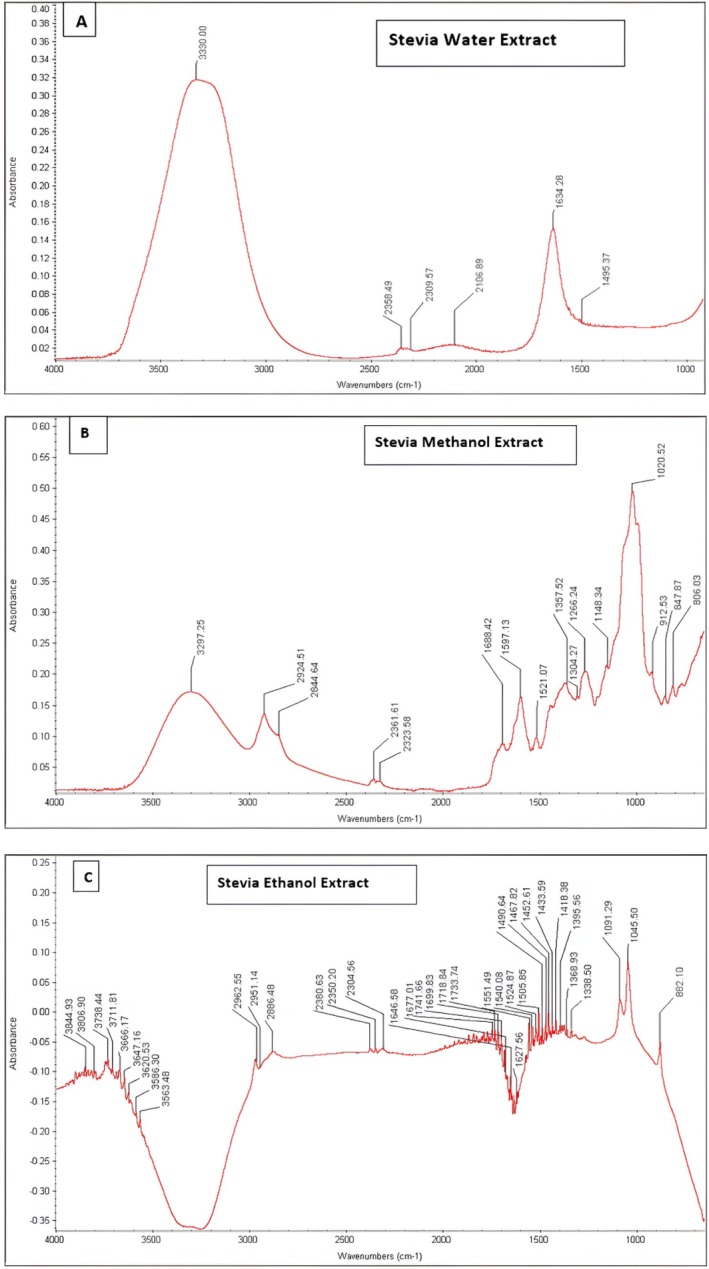
(A–C) FTIR spectra of the pure stevia water, ethanol, and methanol extracts.

The extraordinary influence of stevia extracts with silver and aurum (gold) on the textural properties and morphology of nanoparticles is well visualized by the TEM measurements. Figures 4, 5, 6 show the TEM micrographs of stevia water, methanol, and ethanol extract nanoparticles at 10 nm in AgNO_3_ and AuCl_4_ solutions, respectively. The results unfold the spherical shape of all nanoparticles, and these findings agree with a previous study (Sadeghi et al. 2015), in which they studied a methanolic extract of stevia leaves gold nanoparticles. In the TEM micrographs of methanol extract‐based nanoparticles, agglomeration was observed, indicating less effective stabilization in comparison to water and ethanol extracts. The average particle size was 31.41, 17.82, 24.2, 14.9, 13.20, and 16.27 nm, respectively, in Figure 4A–F.

According to Ali Dheyab et al. (2020), the morphological characterization of AuNPs reveals that the NPs are spherical to semi‐spherical shaped with even geometry. However, our study indicates that among all the stevia water extract of silver nanoparticles, clear round shaped nanoparticles are obtained, whereas the stevia methanol extract showed agglomeration of nanoparticles (Figure 4B). Moreover, the absorption of gold nanoparticles at 515–570 nm by UV–Vis is also confirming its spherical shape (Figure 4D–F). Among all the stevia extracts, silver nanoparticles synthesized from methanol extract exhibited visible agglomeration, which was considered an outlier in terms of uniform size distribution. Additionally, this aligns with the Zetasizer data presented in Table 2, where secondary peaks in the methanol extract (both AgNPs and AuNPs) support the presence of larger aggregates, despite the main peak being used for size analysis. These revisions improve the clarity and completeness of our TEM and size distribution analysis.

### 3.6 Fourier Transform Infrared Spectroscopy (FTIR)

FTIR analyses were used for the characterization of the water, ethanol, and methanol extract‐based silver and gold nanoparticles. The AgNPs and AuNPs synthesized using Stevia leaf extracts beyond centrifugation and microfiltration do not have any additional purification steps. Therefore, the final nanoparticle suspensions may contain not only AgNPs or AuNPs, but also residual phytochemicals from the extracts that may act as capping or stabilizing agents. While FTIR analysis suggests the presence of these compounds on the surface of the nanoparticles, the FTIR spectrum of the stevia leaf extract in water was analyzed, and different bands were obtained. These bands helped in the identification of stevia glycosides (SGS) present in stevia nanoparticles of all extracts. Figure 5A shows the infrared (IR) spectra of silver nitrate solution along with stevia water, ethanol, and methanol extract‐based synthesized AgNPs.

The distinct peak at 3282 cm^−1^ in the AgNO_3_ solution, 3332 cm^−1^ in the SWEAgNPs, 3330 cm^−1^ in the SEEAgNPs, and 3331 cm^−1^ in the SMEAgNPs is showing the stretching of hydroxyl and carboxylic acid groups. The result shows that green synthesized solutions have a peak very close to the silver nitrate solution, which shows, further, the compatibility of stevia water, ethanol, and methanol extract towards the green synthesis of silver solution‐based nanoparticles. FTIR spectra of the pure stevia water, ethanol, and methanol extracts (without metal salts) are given in Figure 6A–C for accurate comparative interpretation.

The two other bands observed in the region between 2000 and 2500 cm^−1^ may correspond to C ≡ C or C ≡ N stretching vibrations, or possibly CO_2_ interference, as reported in the literature (Wong 2015). The third peak in the region between 1500 and 2000 cm^−1^ showed the C=C group's stretching. The clear and distinct band at 1638 cm^−1^ in the AgNO_3_ solution, 1620 cm^−1^ in the SWEAgNPs, 1639 cm^−1^ in the SEEAgNPs, and 1635 cm^−1^ in the SMEAgNPs unfolded the presence of the amide group. These FTIR chromatograms have confirmed the fact that stevia extract has a stronger ability to bind metal, showing that alcohol, secondary amide, alkanes, alkenes, and the amide group, including the protein, could make a layer with the metals of gold and silver that can stabilize the medium and prevent agglomeration (Sadeghi et al. 2015).

According to Figure 5B, the highest peak was noticed at 3301 cm^−1^ in the gold chloride solution, 3339 cm^−1^ in the SWEAuNPs, 3282 cm^−1^ in SMEAuNPs, and 3312 cm^−1^ in SEEAuNPs, which indicates the alcoholic (–OH) and carboxylic acid (–COOH) groups. The results disclose that the green synthesized solutions show a peak very close to the gold chloride solution, which further demonstrates the compatibility of stevia water, ethanol, and methanol extract towards the green synthesis of gold solution‐based nanoparticles. Table 3 shows shifts in O–H and amide bands confirming participation in reduction and capping. The absence of new peaks indicates no major contamination or degradation. The C ≡ C/C ≡ N region (~2100–2200 cm^−1^) may also involve CO_2_ interference or minor phyto compounds. The two other bands of the AuCl_4_ solution were close to the stevia extract‐based nanoparticles between 2000 and 2500 cm^−1^, and this region shows the stretching vibration type and the presence of S‐H and O=C=O groups. The third peak in the region between 1500 and 2000 cm^−1^ showed the C=C group's stretching. The clear and distinct band in 1627–1638 cm^−1^ also showed the presence of an amide group. Similar FTIR‐based profiling for stress‐induced biochemical changes in plant systems has been reported by Bhat et al. (2023, 2022) providing support for the functional group identification and interpretation of spectral shifts observed in this study. FTIR spectra also assure that hydroxyl, carbonyl, and amide groups in Stevia extracts help in the stabilization of nanoparticles. The hydroxyl and carboxyl groups prevent aggregation by formation of coordination bonding with gold and silver nanoparticles, creating the steric barrier and stabilizing organic layer. Colloidal stability is enhanced by electrostatic repulsion from polar functional groups, while amide groups play a significant role via hydrogen bonding and surface capping (Pasieczna‐Patkowska et al. 2025).

**TABLE 3:** Functional group profiling by FTIR of stevia extracts and synthesized AgNPs/AuNPs.

Solvent/Sample	Peak position (cm^−1^)	Assigned functional group	Change after NP synthesis
Water extract	3332	O–H stretch (phenols, alcohols)	Present in SWEAgNPs at 3332; unchanged
	1620	C=O stretch/Amide I (proteins)	Slight shift from 1638 (AgNO_3_ control)
	2120–2160 (broad)	C ≡ C/C ≡ N stretch or CO_2_ interference	Broad band persists; may indicate S–H or CO_2_
SWEAgNPs	3332	O–H stretch	Intensity reduced; involved in binding
	1620	Amide I (C=O, protein)	Peak retained with slight shift
	2160	CO_2_/alkyne/nitrile region	Maintained
SWEAuNPs	3339	O–H, COOH (broad)	Similar band; slight shift from extract
	1635	Amide group	Close to extract; indicates capping
	2160	Same as above	Minor changes
Methanol extract	3330	O–H, N–H stretch	Broad intense peak
	1635	C=O (amide)	Typical protein signal
	2160	Possibly C ≡ C/C ≡ N or CO_2_	Present
SMEAgNPs	3331	O–H stretch	Slightly shifted; peak narrowed
	1635	Amide I	Retained
	2160	—	Unchanged
SMEAuNPs	3282	O–H, N–H stretch	Decrease in intensity
	1638	C=O/Amide	Slight increase in intensity
Ethanol extract	3330	O–H/H‐bonded N–H	Broad and intense
	1639	C=O/Amide	Retained
	2160	Alkyne/nitrile region or CO_2_	Present
SEEAgNPs	3330	O–H stretch	Lower intensity
	1639	Amide I	Sharp; stable
SEEAuNPs	3312	O–H, COOH	Slight shift; suggests involvement
	1627	Amide region	Close to extract

The results display that the synthesis of silver and gold nanoparticles by stevia leaf extracts contains functional groups such as amines, alcohols, ketones, aldehydes, and carboxylic acids. The presence of these functional and secondary compounds played a pivotal role in the synthesis, capping, reduction, and stabilization of AgNPs and AuNPs. It has been stated that proteins and metabolites are vigorously intricate in the reduction of Ag to AgNPs by using plant‐based extracts (Abou‐Arab et al. 2010). These functional groups can also influence targeted binding and biocompatibility and can be utilized in the development of biosensors and in nanomedicine (Diez‐Pascual and Rahdar 2022). The antioxidant potential of stevia‐based nanoparticles enhances their application as food antioxidant films, nanocoatings, and in reducing inflammation (Pandiyan et al. 2023).

The presence of the functional groups suggests their close involvement in the reduction and stabilization of AgNO_3_ and AuCl_4_ to AgNPs and AuNPs, respectively, where the presence of the above‐mentioned functional groups helped in the absorption of compounds on silver and gold NPs. UV–Vis and TEM results support good dispersion, especially in methanol and ethanol extract‐based nanoparticles.

## 4 Conclusion

In this research, water, methanol, and ethanol extracts of *Stevia rebaudiana* were used to synthesize silver (AgNPs) and gold (AuNPs) nanoparticles. This research for the first time demonstrated the efficacy of these extracts' synthesis of nanoparticles as a green technology. Characterization of nanoparticles by UV–VIS spectroscopy confirms the nanoparticles formation, showing SPR at 400–450 nm for AgNPs and 500–550 nm for AuNPs, with further peaks at 650–700 nm. TEM results exhibit spherical, well‐dispersed nanoparticles. The FTIR revealed the presence of functional groups from the stevia extracts responsible for reducing and stabilizing the nanoparticles. The nanoparticles had satisfactory stability, with diverse sizes and polydispersity indices across different solvent extracts. These green‐synthesized nanoparticles offer promising possibilities in the field of food technology as antimicrobial packaging and additives, and in bioengineering; their biocompatibility and functional surface chemistry make them ideal as biosensors, drug delivery systems, and wound healing materials.

Stevia‐mediated nanoparticle synthesis is a cost‐effective, sustainable approach with an industrial standpoint. However, scaling up presents challenges of variability in plant phytochemicals and standardization of optimum procedures. However, advancements in bioprocess engineering and solvent recycling can facilitate commercialization, while integrating Stevia synthesis into existing industries enhances economic viability. Thus, with optimization, this method offers a scalable solution for green synthesis of nanoparticles. Overall, this research underlines the potential of stevia extracts as strong reducing agents for the eco‐friendly and efficient fabrication of silver and gold nanoparticles. Future studies should focus on confirming the role of organic molecules from Stevia extracts as stabilizing Au^3+^ agents through advanced characterization techniques such as X‐ray diffraction (XRD), scanning electron microscopy (SEM), energy‐dispersive X‐ray spectroscopy (EDX), dynamic light scattering (DLS), and zeta potential analysis. Additionally, thermal stability can be assessed via thermogravimetric analysis (TGA) and differential scanning calorimetry (DSC), and biological safety should be evaluated through comprehensive cytotoxicity assays.

## Author Contributions

**Muhammad Farhan Jahangir Chughtai:** conceptualization (equal), data curation (equal), formal analysis (equal), investigation (equal), methodology (equal), software (equal), visualization (equal), writing – original draft (equal). **Samreen Ahsan:** formal analysis (equal), investigation (equal), methodology (equal). **Adnan Khaliq:** methodology (equal), validation (equal). **Muhammad Adil Farooq:** data curation (equal), formal analysis (equal). **Mir Muhammad Nasir Qayyum:** writing – review and editing (equal). **Atif Liaqat:** methodology (equal), resources (equal), validation (equal). **Saira Tanweer:** validation (equal), writing – review and editing (equal). **Tariq Mehmood:** data curation (equal), investigation (equal), writing – review and editing (equal). **Muhammad Zubair Khalid:** conceptualization (equal), validation (equal), writing – review and editing (equal). **Waseem Khalid:** validation (equal), writing – review and editing (equal). **Yassine Jaouhari:** validation (equal), writing – review and editing (equal). **Matteo Bordiga:** data curation (equal), investigation (equal), writing – review and editing (equal). **Suleiman A. Althawab:** writing – review and editing (equal). **Tawfiq Alsulami:** funding acquisition (equal), supervision (equal), writing – review and editing (equal).

## Conflicts of Interest

The authors declare no conflicts of interest.

## Data Availability

The data that support the findings of this study are available on request from the corresponding author.
